# Fabrication of Implant-Supported Auricular Prosthesis Using Artificial Intelligence

**DOI:** 10.7759/cureus.60267

**Published:** 2024-05-14

**Authors:** Ankita Pathak, Mithilesh M Dhamande, Smruti Gujjelwar, Pritam Das, Ekta V Chheda, Rahul Puthenkandathil

**Affiliations:** 1 Prosthodontics, Sharad Pawar Dental College and Hospital, Datta Meghe Institute of Higher Education and Research, Wardha, IND; 2 Prosthodontics, Kalinga Institute of Medical Sciences, Bhubaneshwar, IND; 3 Prosthodontics, Government Dental College and Hospital, Ahmedabad, IND; 4 Prosthodontics and Crown and Bridge, AB Shetty Memorial Institute of Dental Sciences, Mangalore, IND

**Keywords:** implant-supported auricular prosthesis, artificial intelligence, maxillofacial prosthesis, ear injury, rta-road traffic accidents

## Abstract

The absence of any organ of the facial region causes an asymmetrical appearance. This asymmetrical appearance can cause social dilemmas for the patient. The maxillofacial technician, the prosthodontist, and the patient must work closely together to fabricate an epithesis. On the implants, a superstructure is first constructed. Most of it is made up of rings and a bar that joins the implants. The firm acrylic resin base of the epithesis is equipped with clips that serve as the epithesis's retention mechanism. The actual epithesis is made of silicone rubber. The epithesis has to be shaped and colored with extreme caution. An appropriate substitute is an auricular prosthesis that is implant-retained. Microtia, deformity, malformation, and loss of the external ear, either partially or completely, can result from a variety of inherited genetic conditions. To evaluate the symmetry of both ears, artificial intelligence (AI) software is used. An Instagram lens Gridset by crystalwavesxx was used to correct and verify the bilateral symmetry of the patient. This case report primarily focuses on the fabrication of implant-supported auricular prostheses using AI.

## Introduction

One major cosmetic issue is the lack of an external ear. Because of the complicated structure of the ear, fabricating an auricular prosthesis is a laborious process [1]. Although autogenous grafts are the best option for treatment, prosthetic replacement is an excellent alternate option for individuals in whom autogenous graft repair is not feasible [2]. To camouflage the asymmetry of the face due to maxillofacial defect that often causes a social stigma as a result of a distorted physical appearance, maxillofacial prosthesis is the ultimate choice of treatment [3].

Even after treatment, a patient from a road traffic accident (RTA) may have a serious paralyzing handicap. Retention is the primary factor that determines the maxillofacial prosthesis' long-term success [4]. A cosmetic prosthesis secured by implants incorporated into the skull may be able to successfully replace a severely distorted external ear. Nowadays, using such implants is a widely accepted way to get a consistent outcome in maxillofacial rehabilitation. This case study details an implant-supported silicone prosthesis-based safe, easy, and affordable rehabilitation technique for a patient who has lost his right auricle. Implants were placed in the mastoid region of the temporal bone. An auricular silicone prosthesis was used for ear reconstruction, and it was secured with a header bar and clip attachment [4,5].

Along the surgical excision, the major concern has always been aesthetics. Rehabilitation with auricular prosthesis restores the patient's aesthetics and cosmetic appearance [6-8]. For the symmetrical natural-looking appearance, artificial intelligence (AI)-generated software was used. The life-like appearance of the prosthesis boosts the patient's confidence and improves their psychological mindset. The following case report describes an innovative technique for the fabrication of implant-supported auricular prosthesis using AI-generated software application.

## Case presentation

A 34-year-old male patient reported to the Department of Maxillofacial Prosthodontics with the chief complaint of an asymmetric and discolored auricular prosthesis in the right mastoid region. Ten years ago, the patient was asymptomatic until he met with RTA and was subsequently hospitalized. Surgical resection of the ear was performed, followed by the placement of implants in a two-stage procedure. After a healing period of six months, an auricular prosthesis was fabricated. Over the next two to three years, two additional prostheses were manufactured due to concerns regarding color, size, and symmetry, yet none met the patient's expectations.

**Figure 1:**
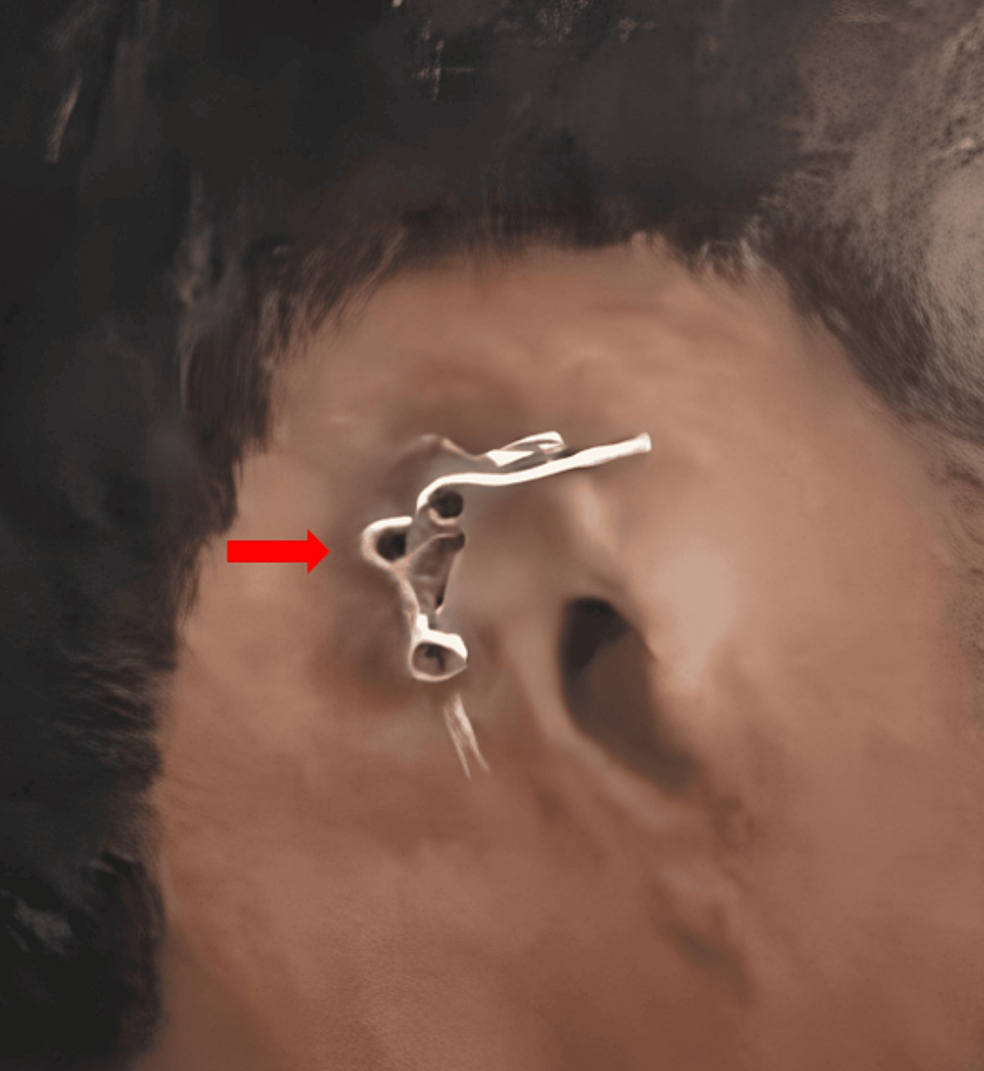
Implants in the mastoid region with Hader clip attachment

Upon inspection, complete surgical removal of the external ear, with two implants featuring a Hader bar attachment, was observed. The surrounding skin exhibited no signs of inflammation, and physical examination revealed no pain upon palpation as shown in Figure 1.

**Figure 2:**
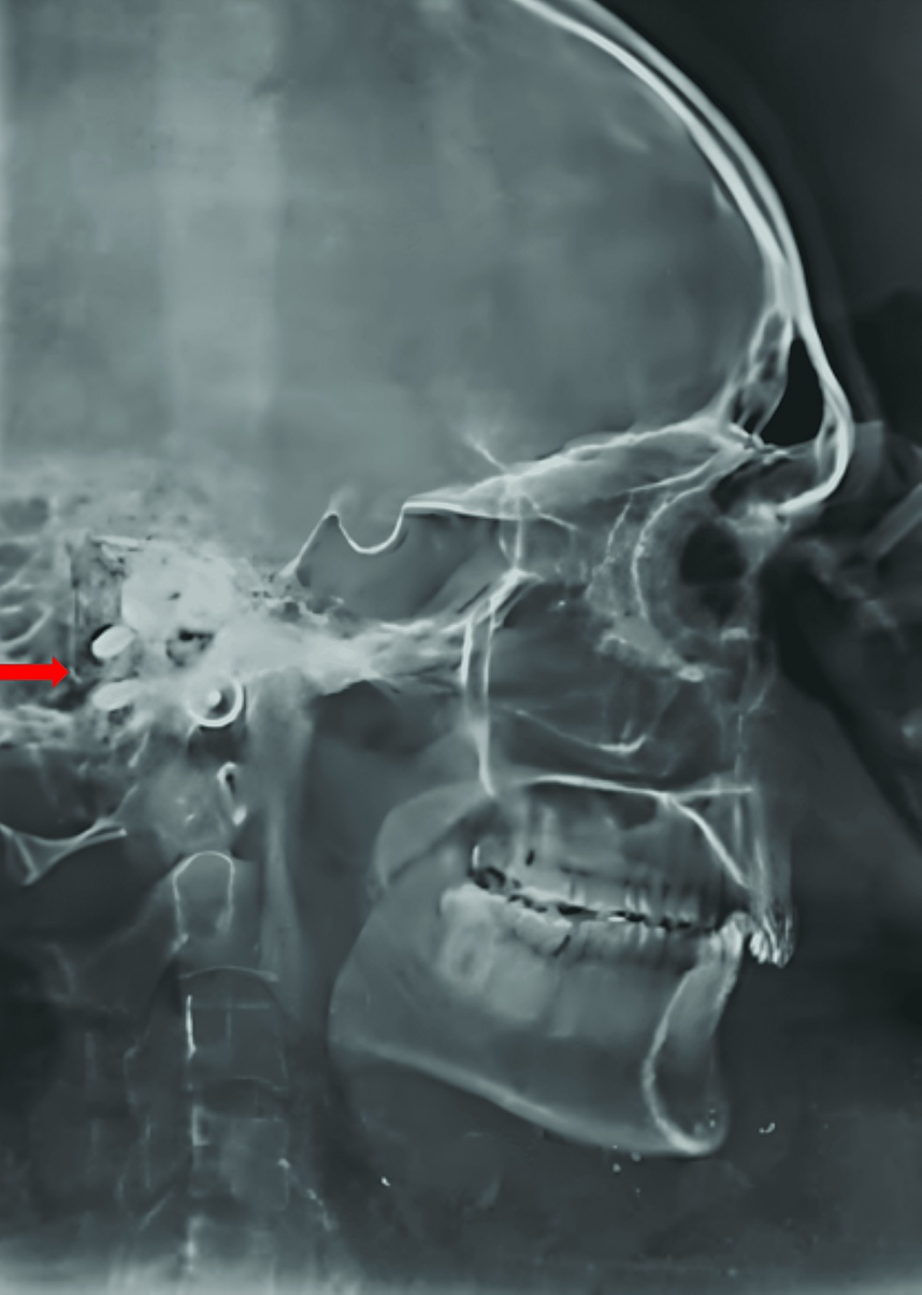
Lateral cephalogram showing implants in the mastoid region

Implant stability was assessed, and a radiographic examination was conducted using a lateral cephalogram as shown in Figure 2.

**Figure 3:**
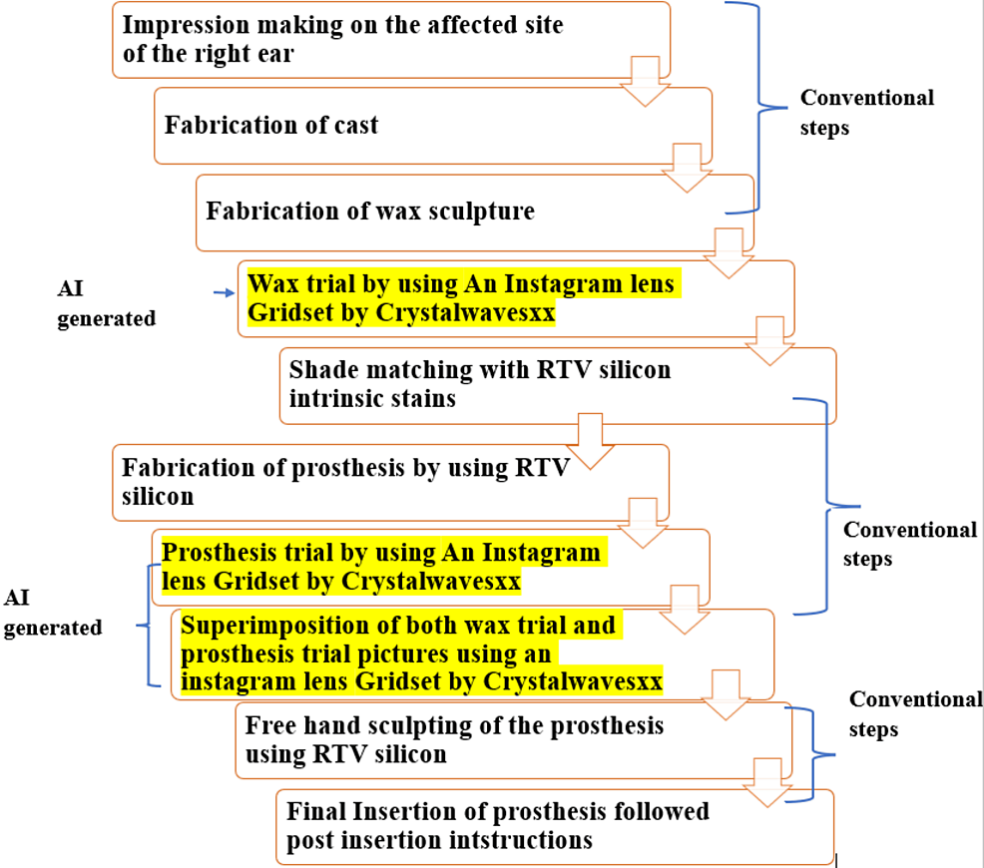
A step-by-step protocol for the fabrication of auricular prosthesis with a confluence of conventional and AI-generated technique AI: artificial intelligence; RTV: room-temperature vulcanizing

After a thorough examination, it was determined that a new auricular prosthesis would be fabricated using an innovative AI-generated application technique. A step-by-step protocol is elaborated in Figure 3.

**Figure 4:**
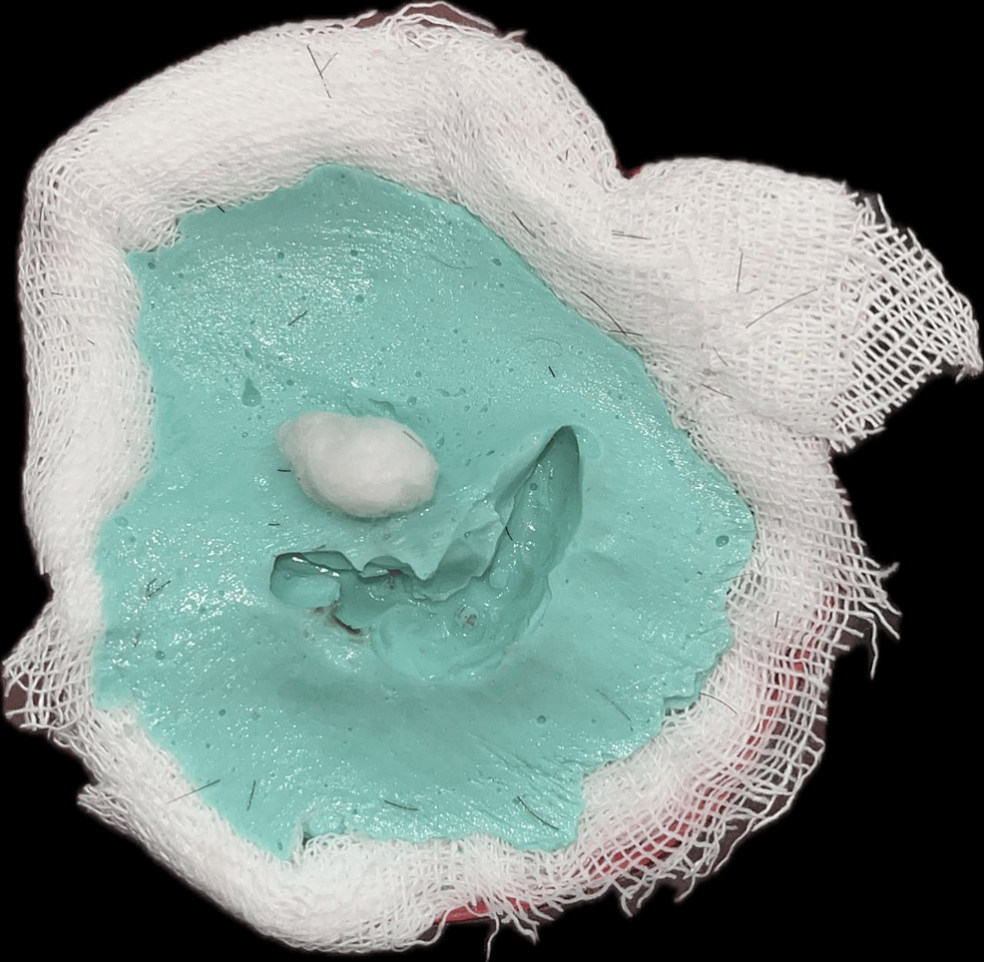
Impression of the affected site

**Figure 5:**
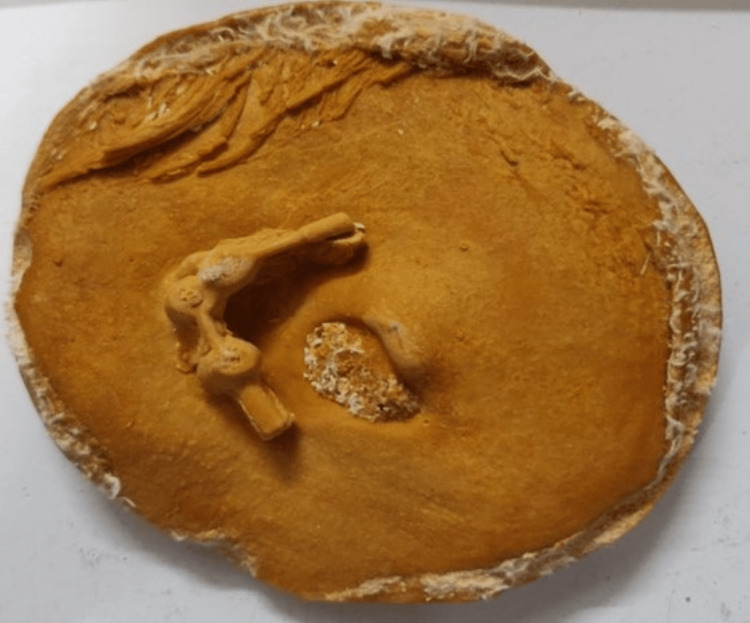
Fabricated cast

To accurately replicate the exact anatomy, both the deformed and normal site impressions were conventionally made. Petroleum jelly was applied around the mastoid region, and an impression was taken using irreversible hydrocolloid (Prime Chrome Chromatic Alginate Impression Material, Prime Dental Products Pvt Ltd, Thane, India), with dental plaster used over alginate for added strength. The impression was poured into dental stone type 4, and cast fabrication was completed as depicted in Figure 4 and Figure 5.

**Figure 6:**
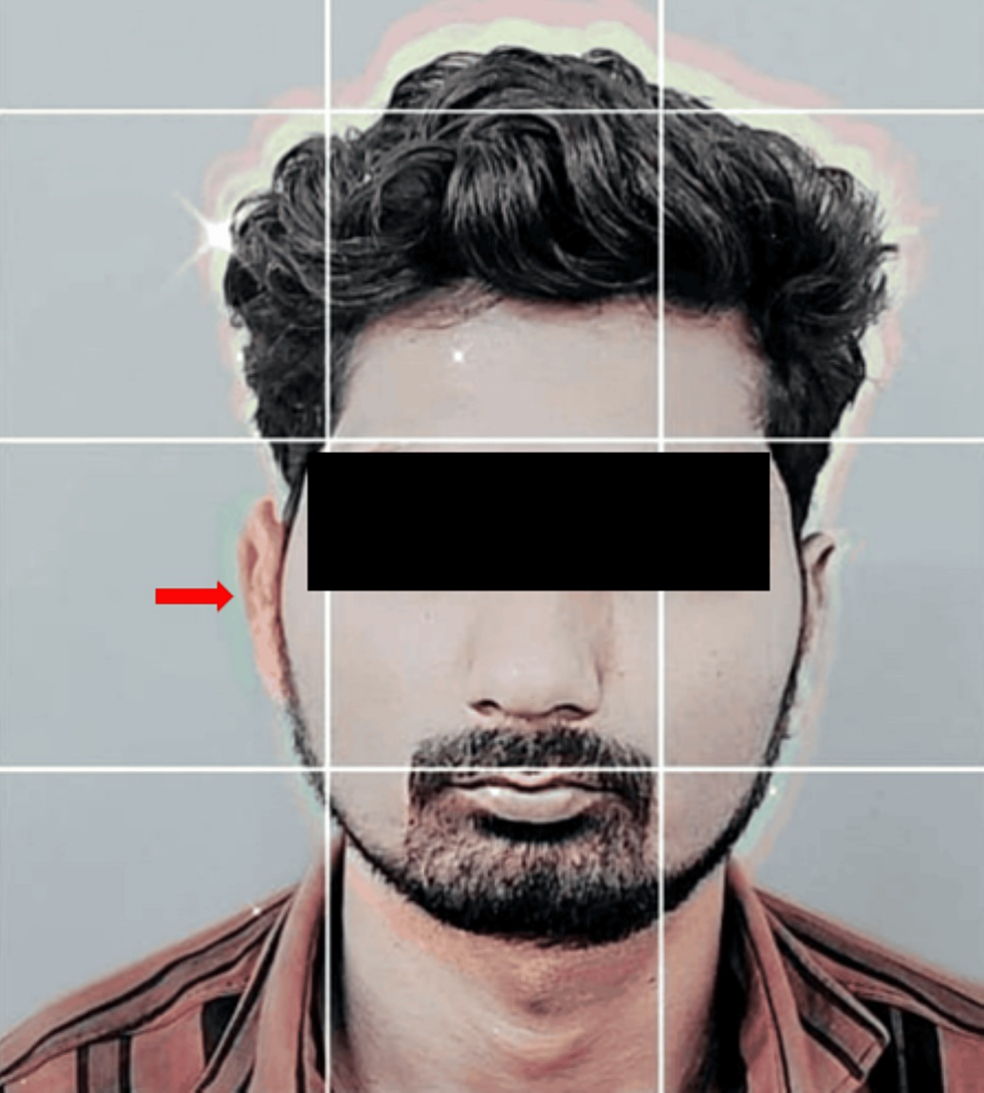
Gridset lens by crystalwavesxx for wax trial evaluating symmetry

A wax sculpture was then meticulously carved using modeling wax (Maarc Modelling Wax, Dental Genie, New Delhi, India), with careful reference to the contralateral left ear to ensure precise replication of the anatomy. Subsequently, a wax trial was conducted to assess the symmetry. Utilizing the Instagram application in story mode, Gridset by crystalwavesxx lens scanned the patient's face upright to aid in the symmetry assessment. The wax pattern was adjusted on the patient's face to achieve optimal symmetry, with the grid pattern from the filter superimposed for reference and the resulting image saved in the gallery. A horizontal line just above both ears was taken as a virtual reference plane for the assessment of symmetry. By using this virtual reference plane, bilateral symmetry was established. Figure 6 shows a wax trial using a Gridset lens.

**Figure 7:**
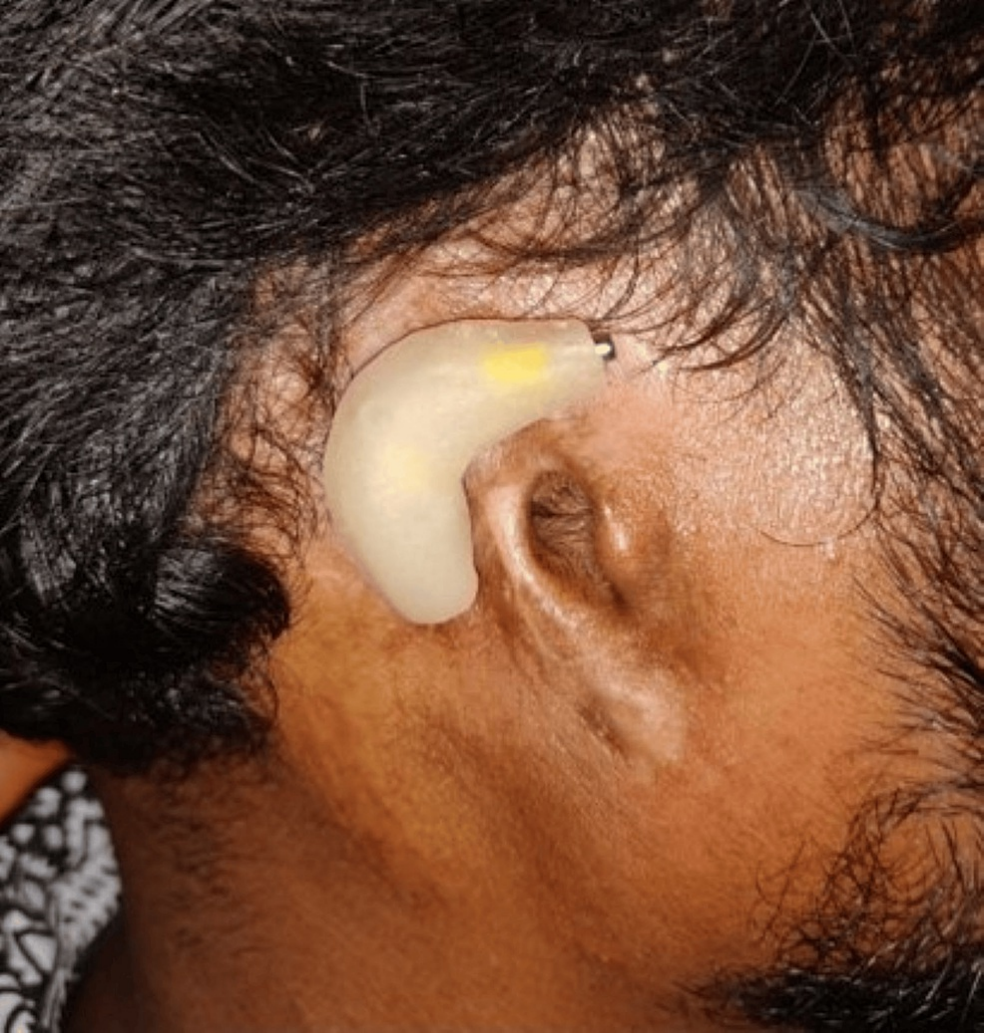
Acrylic stent

Undercuts were blocked, and an acrylic stent was fabricated on the cast for clip attachment in the bar as shown in Figure 7.

**Figure 8:**
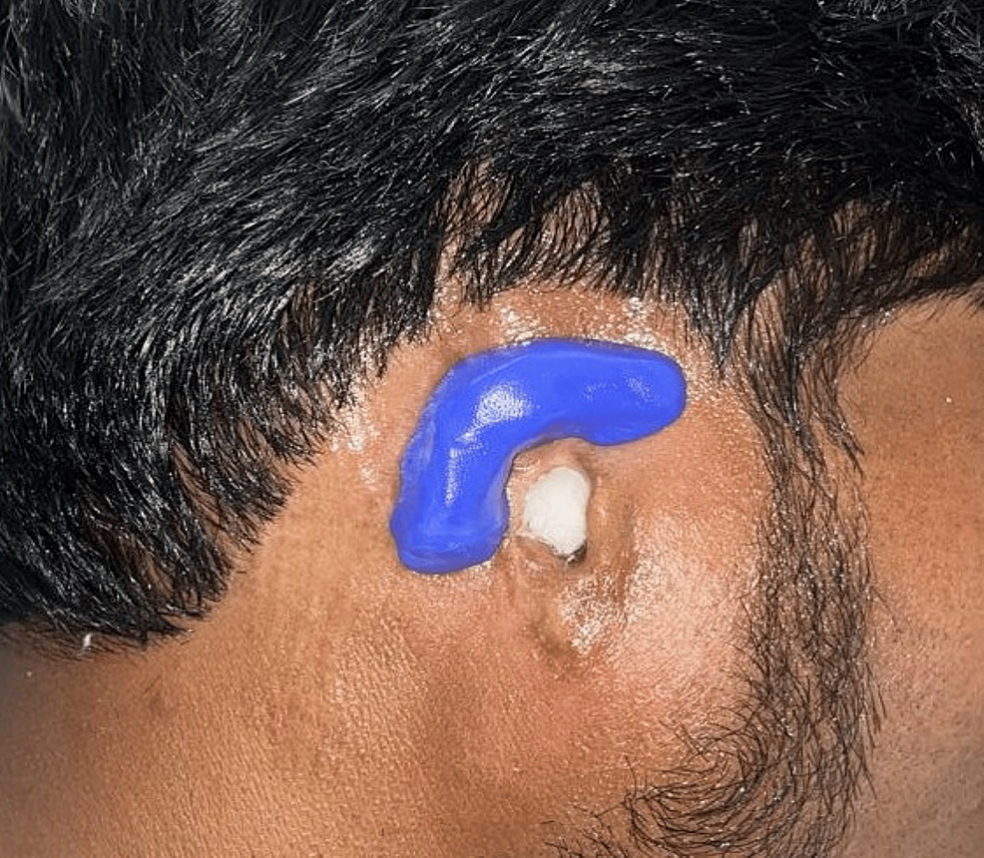
Putty adapted over acrylic stent

A putty index of the acrylic stent was then created and utilized for the final fabrication of the acrylic stent as depicted in Figure 8.

**Figure 9:**
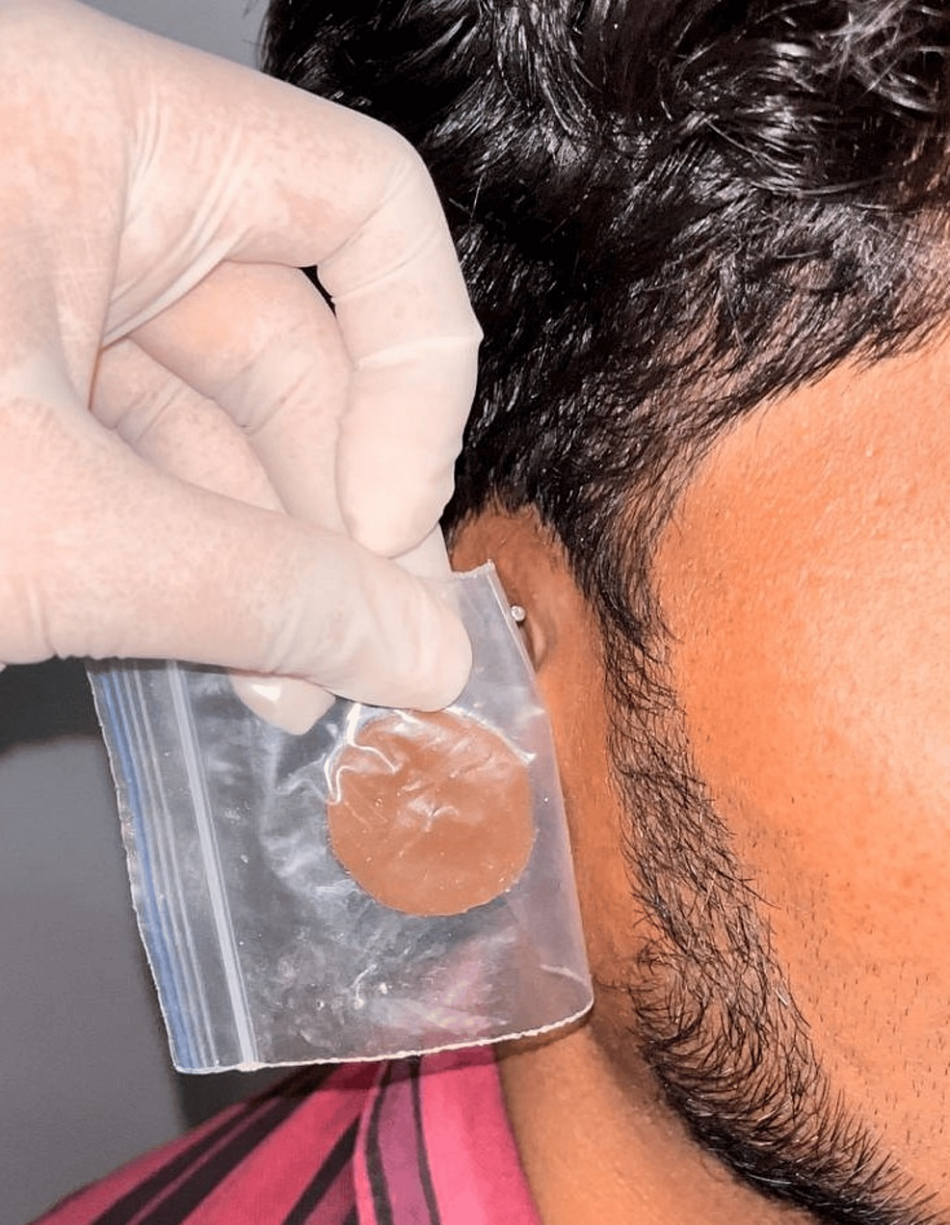
Shade matching

This acrylic stent was adapted to the inner side of the prosthesis to serve as the retention system for the Hader bar and clip attachment. After the patient approved the wax sculpture, shade matching was done with a room-temperature vulcanizing (RTV) silicone (Technovent Silicone Kit M511 Platinum Silicone, Technovent Ltd, Bridgend, UK) during the day (Figure 9).

Intrinsic pigments such as white (P105), yellow (P106), brown (P108), bright red (P112), black (P109), and flocking powder in red (P301) and purple (P302) shades (intrinsic and extrinsic pigments, Technovent Silicone Kit M511 Platinum Silicone) were carefully used to mimic the patient's natural skin texture. The acrylic stent that was placed on the Hader bar was then replicated using a putty index. Using Technovent's RTV silicone, a cast made from this imprint was used to fabricate the prosthesis. After fabrication, the prosthesis was retrieved from the mold and allowed to cool in a hot air oven.

**Figure 10:**
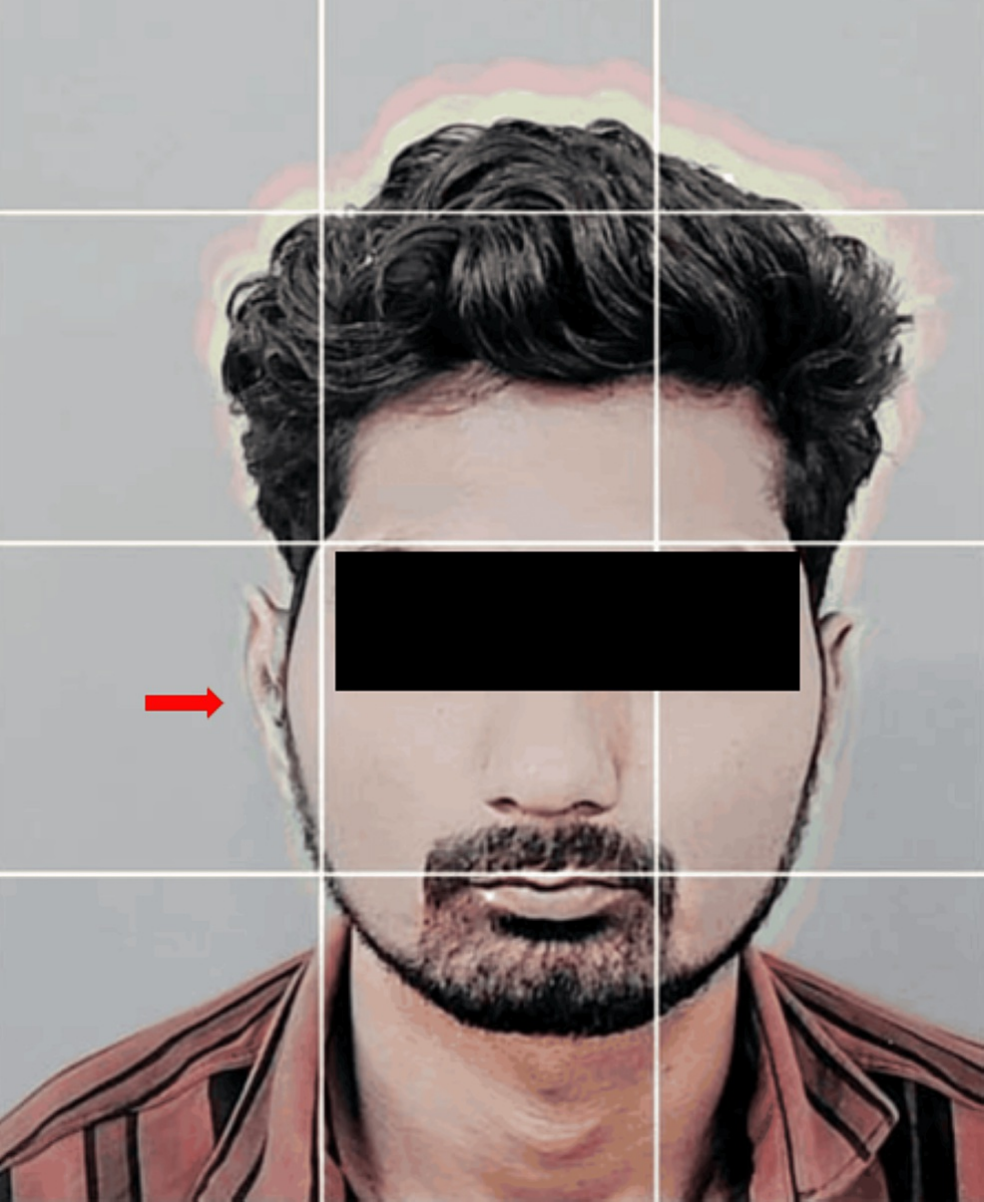
Gridset lens used for final prosthesis insertion

Upon retrieval, the prosthesis was adapted to the patient, and the same lens was utilized to assess symmetry, with the resulting picture of the silicone prosthesis saved in the gallery. The previously used virtual reference plane was used again on the final prosthesis during insertion. The wax trial picture and the inserted prosthesis picture were superimposed, allowing for any necessary freehand sculpting and adjustments to achieve bilateral symmetry (Figure 10).

Finishing and polishing burs for the prosthesis ensured a seamless blend with the contralateral ear and concealing margins to the surrounding tissue. After applying extrinsic staining to match the natural skin tone, the stains were sealed using the extrinsic sealant. The space that was made in the prosthesis was then filled with an acrylic stent attached with a clip. Upon placement, prosthesis insertion was performed, with post-insertion instructions provided for hygiene maintenance. Table 1 depicts post-insertion care and commonly faced problems after the insertion of the prosthesis.

**Table 1:** Post-insertion care and commonly faced problems after the insertion of the prosthesis

Post-insertion care	Commonly faced problems
Cleaning with water and neutral soap along with chlorhexidine	Discoloration and deterioration
Cleaning of adjacent tissues is also recommended	Fungal infection and foul smell
Removal before sleeping	Loss of retentive elements and marginal tear

**Figure 11:**
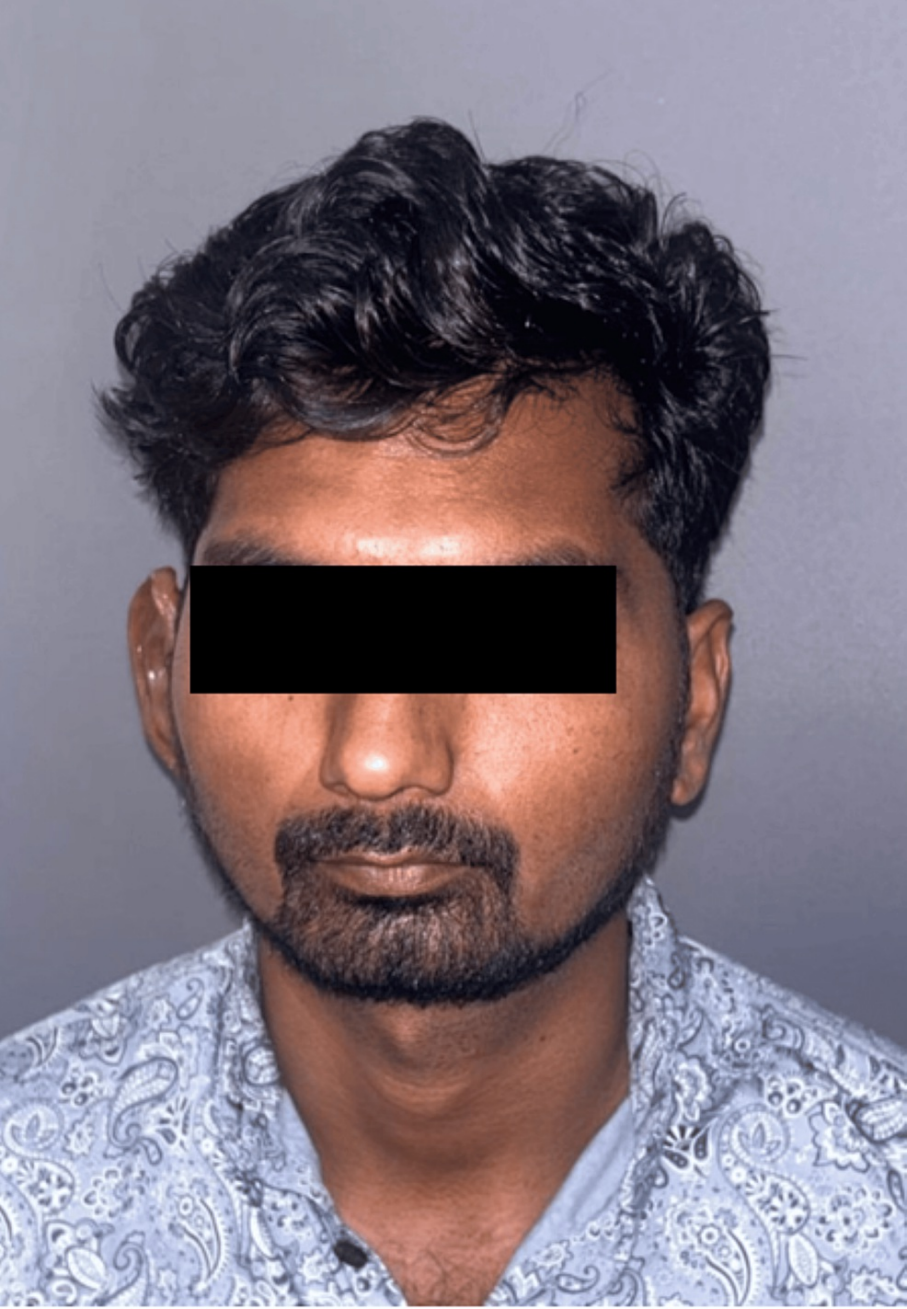
Final prosthesis insertion

A follow-up schedule of six months was arranged to ensure the stability of the retentive component. Following insertion, the patient expressed high satisfaction and contentment with the outcome. Figure 11 shows the inserted prosthesis in the right missing ear region.

## Discussion

A person with significant psychological problems associated with facial defects might avoid social interactions. Considering this, maxillofacial rehabilitation's main objective is to treat cosmetic concerns. Anatomic undercuts, skin adhesives, and implants are essential elements in guaranteeing proper retention. Extraoral implant-retained prostheses are a dependable therapeutic option for maxillofacial rehabilitation [9,10].

Gridset by crystalwavesxx lens in iPhone scanned the patient's face in the upright position, meticulously capturing every contour and feature with precision. The resulting data provided a detailed blueprint for sculpting a symmetrical auricular prosthesis that seamlessly blended with the patient's natural anatomy. A horizontal line just above both ears is considered a virtual reference plane. This reference plane was used at the wax trial and the final insertion of the prosthesis, by superimposing the images. Further, freehand sculpting with RTV silicone was carried out to match the symmetry of the left ear. With advanced technology and skilled prosthodontists, the prosthesis is seamlessly integrated into the patient's appearance. Gridset by crystalwavesxx lens, an advanced AI-driven photographic imaging application in Instagram, scanned the patient's face in the upright position with unparalleled precision. The AI efficiently processed large amounts of data in real time, ensuring every detail of the patient's facial structure was captured accurately, resulting in a precise digital model. The limitation of the above-mentioned innovative technique is that only bilateral symmetry can be assessed, though the cost is not a factor as this application is readily available. The discoloration of the medical-grade silicone used in the fabrication of maxillofacial prostheses occurs over time due to continuous environmental exposure [11]. Thus, the prosthesis needs to be changed regularly. When used to maintain extraoral prostheses like ears, craniofacial implants offer excellent support and retention capabilities as well as improve a patient's appearance and quality of life [12].

Traditional techniques depend upon the artistic dexterity of the clinician or technicians. It is also a tedious job to replicate the tortuous anatomy of the ear. Most of the time, fabricating such prostheses with conventional methods is a bit time-consuming. However, in the presented case, by using an AI-generated application, bilateral symmetry from the frontal view is very well achieved, which was the main concern of the patient. To mitigate the drawbacks of conventional approaches, rapid prototyping techniques for extraoral prostheses have recently been developed. Rapid prototyping is a strategy that may streamline the process and reduce the amount of laboratory work needed. It eliminates the need for prosthodontists to fabricate the wax patterns and tedious impression procedures. High cost is the major limitation for fabricating such a prosthesis. Soon, this method will become the gold standard for fabricating maxillofacial prostheses [13]. Tissue engineering for the treatment of microtia and 3D printing of silicone prostheses are recent developments in maxillofacial prostheses. Further study in this field is required since the material utilized in the 3D printing of silicone prostheses is not true silicone and its biocompatibility is under doubt [14]. Conversely, a set of high-density functionally dissociated cells is utilized onto synthetic biocompatible, biodegradable polymers and then transplanted into an animal model to form functional tissue [15]. Tissue engineering studies performed in animal models have opened up a wide range of potential therapeutic applications [16-20].

## Conclusions

The combination of AI and implant-supported prosthesis fabrication has transformed facial reconstruction, especially in replicating the intricate tortuous and convulsive anatomy of the ear. AI algorithms ensure precise replication through detailed scanning and analysis, restoring symmetry and individuality to patients' features. Social platforms like Instagram also play a role, allowing people to share experiences and highlight the transformative technology. This blend of innovation and compassion helps individuals embrace their identities, boosting confidence and dignity in challenging times.
